# Supplementary material for: Early-life exposure to low-dose oxidants can increase longevity via microbiome remodelling in Drosophila
Source: Nat Commun. 2018 Mar 7;9:975. doi: 10.1038/s41467-018-03070-w (PMC5841413; doi:10.1038/s41467-018-03070-w)
Supplement: Supplementary file 2 — Supplementary Information [file 41467_2018_3070_MOESM2_ESM.pdf]

Early-life exposure to low-dose oxidants can increase longevity via microbiome remodelling in *Drosophila*

Obata et al.

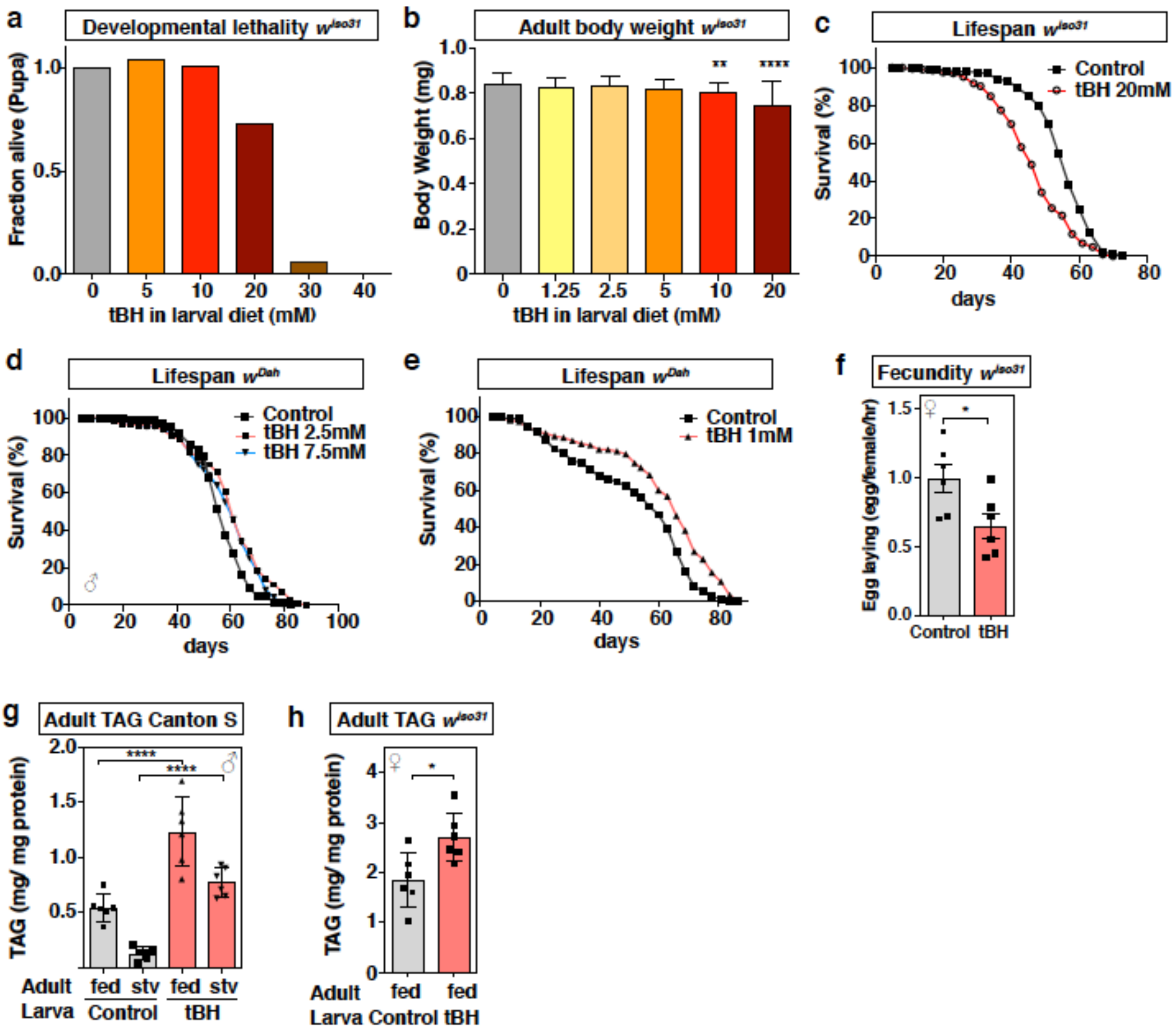

Supplementary Figure 1, Low-dose tBH in the larval diet decreases fecundity but increases lifespan. a, Fraction of w^{iso31} larvae exposed to different tBH concentrations (0-40mM) in standard diet that survive until pupariation (n>200). **b,** Adult body weights of w^{iso31} male flies raised on different tBH concentrations in the standard diet. Mean ± SEM (n=50). **c,** Lifespan of w^{iso31} male flies raised on 20mM tBH. **d,e,** Lifespan of w^{Dah} male (**d**) or female (**e**) flies raised on 1-7.5mM tBH. **f,** Fecundity of w^{iso31} female flies raised on 5mM tBH. Mean ± SEM (n=6). **g,h,** Whole body TAG (mg/mg of protein) in Canton S male (**g**) or w^{iso31} female (**h**) flies raised on 5mM tBH with or without 24-hour starvation. Mean ± SEM (n=6). Asterisks indicate *$p<0.05$, **$p<0.01$, ****$p<0.0001$, see Materials and Methods for details of statistical tests used in this and subsequent all figures. Statistics for lifespan curves are shown in Supplementary Table 1.

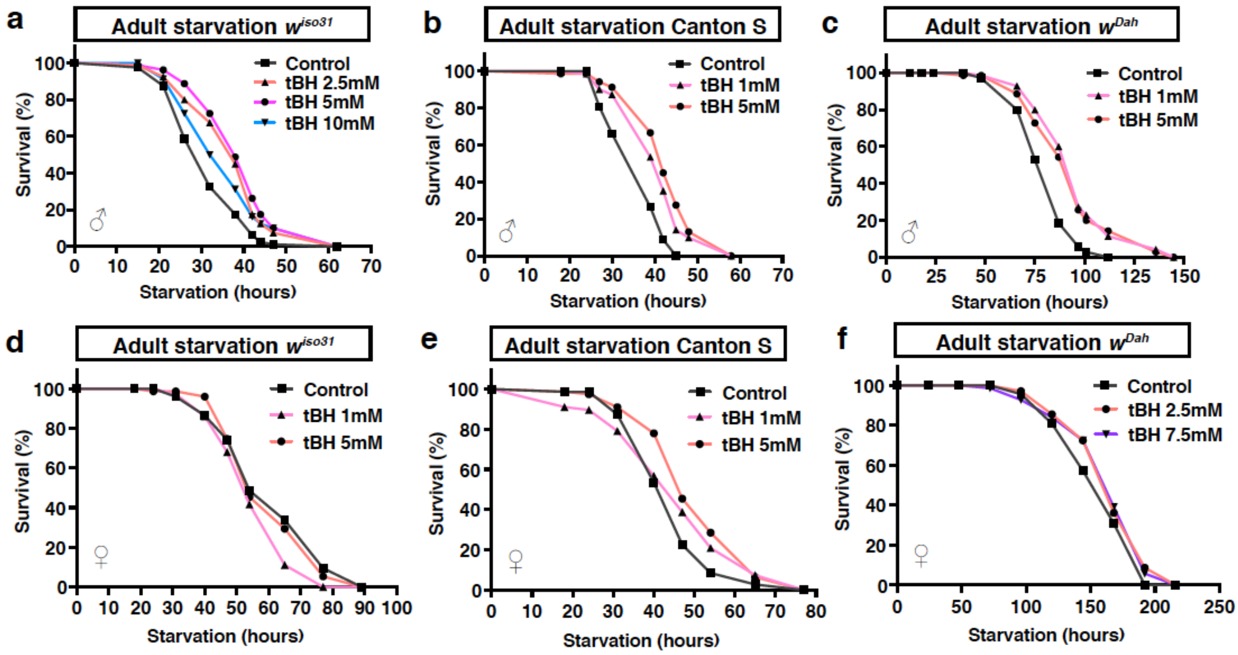

Supplementary Figure 2, tBH-experienced flies are resistant to starvation stress. a-f, Starvation survival curves for w^{iso31} (**a**,**d**), Canton S (**b**,**e**), and w^{Dah} (**c**,**f**) flies on PBS/agar medium. Either male (**a-c**) or female (**d-f**) flies were used. Statistics for survival curves are in Supplementary Table 1.

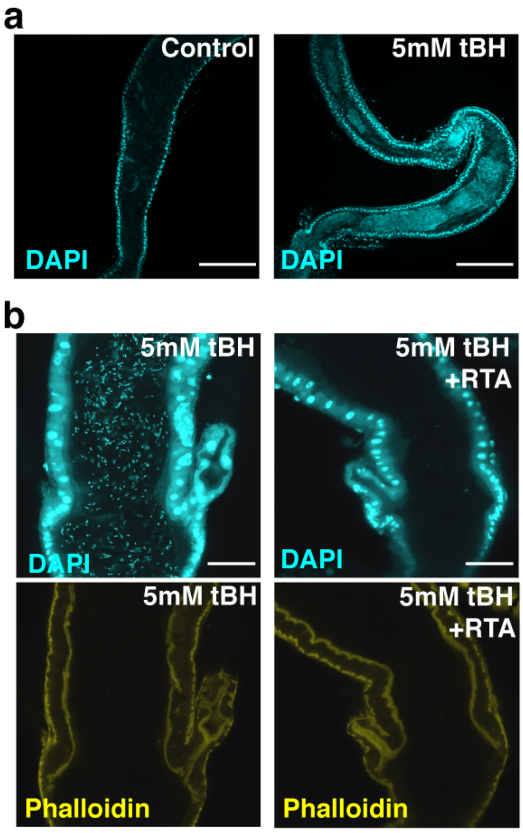

Supplementary Figure 3, Larval tBH exposure increases DAPI⁺ bacteria in the adult gut. **a,** Representative image of DAPI staining of the adult w^{iso31} male gut. Scale bar, 200 µm. **b,** Images of DAPI and phalloidin staining of w^{iso31} adult male at midgut/hindgut junction after 6 days on standard diet without (left) or with (right) the RTA antibiotic cocktail (Rifamycin, Tetracycline, and Ampicillin). Scale bar, 50 µm.

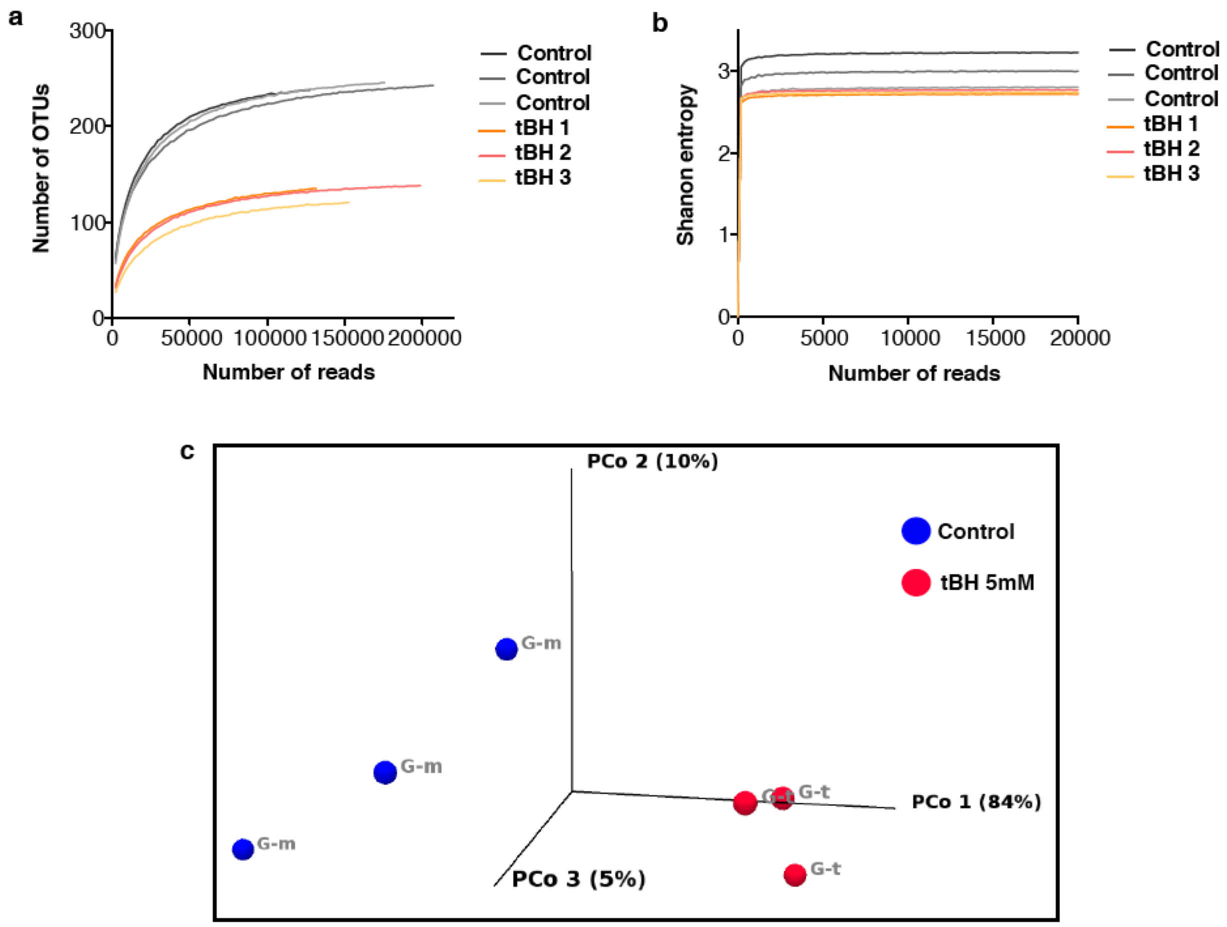

Supplementary Figure 4, Gut microbiome diversity indices of control versus tBH-experienced flies. Rarefaction curves (a), Shannon entropy (b) and PCo plot of Bray-Curtis dissimilarity (**c**) for triplicate samples from control or tBH-experienced flies.

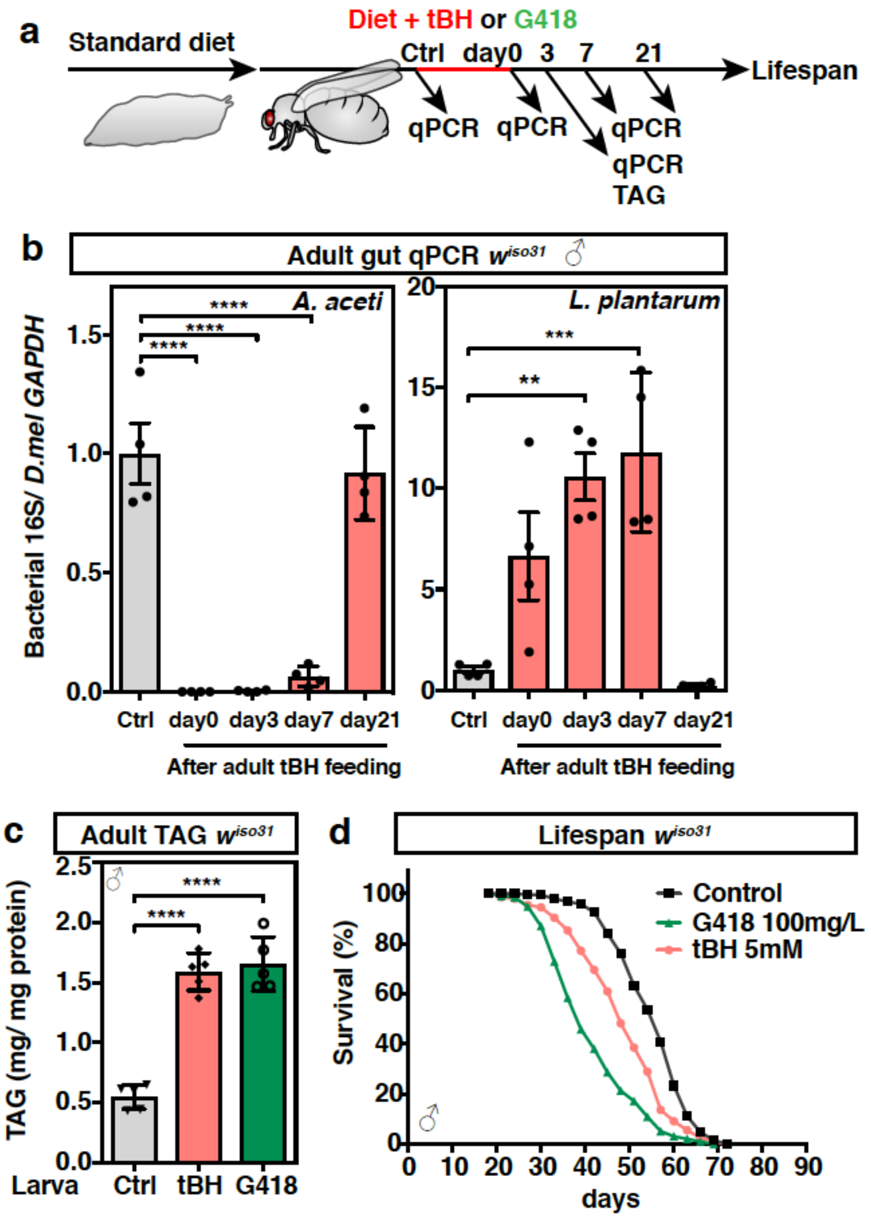

Supplementary Figure 5, Adult onset tBH does not stably deplete *A. aceti* or extend lifespan but it does increase TAG storage. a, Outline of experimental strategy. **b,** Quantitative PCR analysis of bacteria from w^{iso31} adult male guts using species-specific primers. Adult male flies were fed 5mM tBH-containing diet for 6 days and then transferred to standard diet for a further 0, 3, 7 or 21 days before analysis. Control (Ctrl) samples were collected prior to the start of tBH exposure as shown in A. Mean ± SEM (n=4). **c,** Whole body TAG (mg/mg of protein) in w^{iso31} male flies 3 days after a 6-day exposure to G418 (100mg/L) or tBH (5mM) as shown in a. Mean ± SEM (n=5). **d,** Lifespan of w^{iso31} male flies exposed as adults to G418 or tBH for 6 days. Asterisks indicate **p<0.01, ***p<0.001, ****p<0.0001. Statistics for survival curves are shown in Supplementary Table 1.

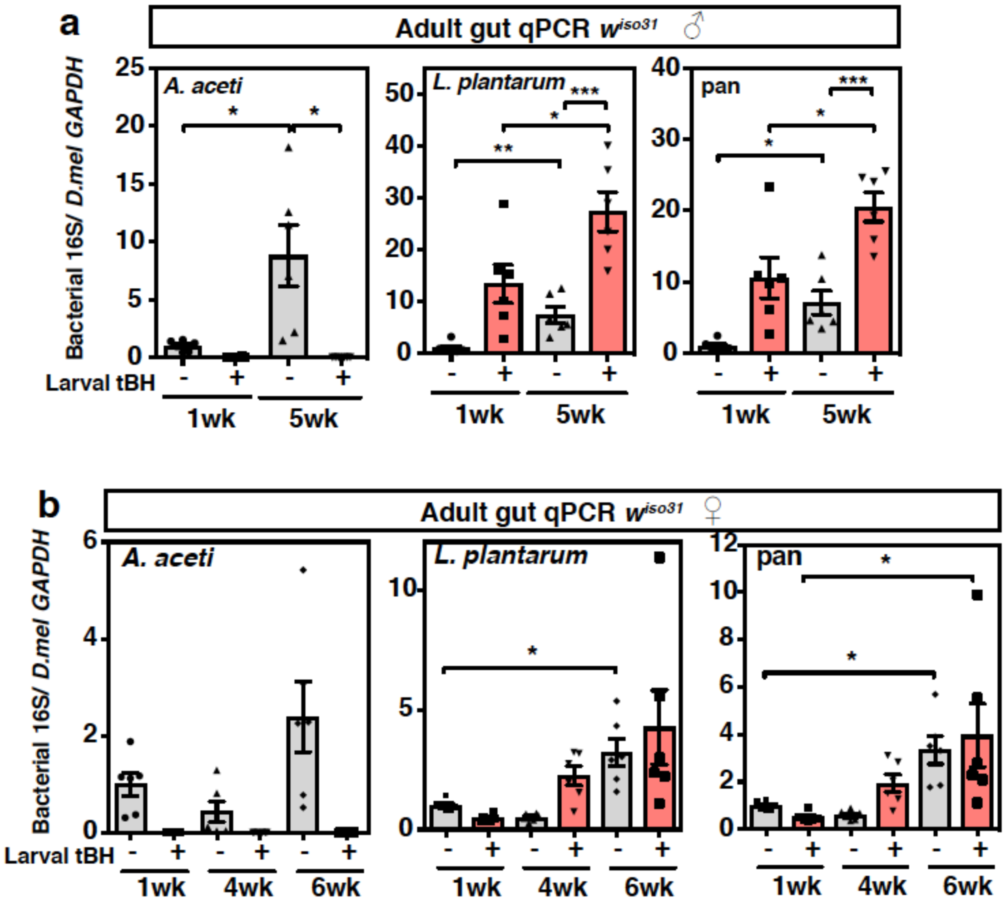

Supplementary Figure 6, tBH-experienced flies show an age-related increase in gut bacteria but life-long depletion of *A. aceti*. a,b, Quantitative PCR analysis of bacteria from the adult gut of w^{iso31} male **(a)** or female **(b)** flies of various ages using species-specific or pan primers. Mean ± SEM (n=6). Asterisks indicate *p<0.05, **p<0.01, ***p<0.001.

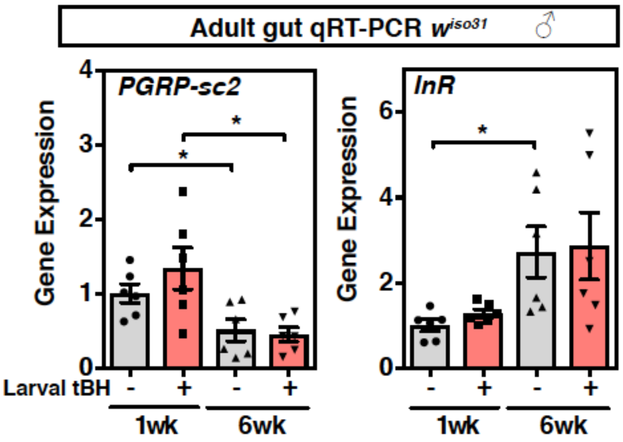

Supplementary Figure 7, Larval tBH does not inhibit age-related dFoxO activation in the adult gut. Quantitative RT-PCR analysis of the dFoxO targets *PGRP-sc2* and *InR* in the gut of young (1week) or old (6week) w^{iso31} male flies. Mean ± SEM (*n*=6). Asterisks indicate $*p<0.05$.

Supplementary Table 1, Statistics for survival curves. Cohort sizes, mean and median lifespans, percentage changes, and log-rank tests for Kaplan-Meier survival curves in this study.

Fig.1b	*n*	mean(% change)	median(% change)	Log-rank (vs control)
Control	192	59.49	59	-
tBH1.25mM	205	70.4 (+18.3%)	71 (+20.3%)	p<0.0001
tBH2.5mM	202	66.66 (+12.1%)	68 (+15.3%)	p<0.0001
tBH5mM	207	71.2 (+19.7%)	71 (+20.3%)	p<0.0001
tBH10mM	200	69.83 (+17.4%)	71 (+20.3%)	p<0.0001

Fig.1c	*n*	mean(% change)	median(% change)	Log-rank (vs control)
Control	196	59.6	62	-
tBH1mM	127	69.58 (+16.7%)	71 (+14.5%)	p<0.0001
tBH5mM	200	66.67 (+11.9%)	71 (+14.5%)	p<0.0001

Fig.1d	*n*	mean(% change)	median(% change)	Log-rank (vs control)
Control	195	42.45	43	-
tBH5mM	162	54.5 (+28.4%)	55 2 (+27.9%)	p<0.0001

Fig.1e	*n*	mean(% change)	median(% change)	Log-rank (vs control)
Control	191	40.52	44	-
tBH1mM	124	49.71 (+22.7%)	56 (+27.3%)	p<0.0001
tBH5mM	196	49.69 (+22.6%)	59 (+34.1%)	p<0.0001

Fig.1f	*n*	mean(% change)	median(% change)	Log-rank (vs control)
Control	191	52.77	54	-
PQ1mM	170	59.22 (+12.2%)	60 (+11.1%)	p<0.0001

Fig.S1c	*n*	mean(% change)	median(% change)	Log-rank (vs control)
Control	200	55.06	57	-
tBH20mM	206	46.47 (+15.6%)	46 (-19.3%)	p<0.0001

Fig.S1d	*n*	mean(% change)	median(% change)	Log-rank (vs control)
Control	198	56.69	58	-
tBH2.5mM	212	60.07 (+6.0%)	61 (+5.2%)	p<0.0001
tBH7.5mM	204	58.57 (+3.3%)	61 (+5.2%)	p=0.0011

Fig.S1e	*n*	mean(% change)	median(% change)	Log-rank (vs control)
Control	139	52.18	60	-
tBH 1mM	134	60.93 (+16.8%)	66 (+10.0%)	p<0.0001

Fig.2d	*n*	mean(% change)	median(% change)	Log-rank (vs control)
Control	79	4.32	5	-
tBH2.5mM	80	4.33 (0.2%)	5 (0%)	p=0.8983
tBH5mM	80	4.06 (-6.0%)	4 (-20%)	p=0.2338
tBH10mM	80	3.84 (-11.1%)	4 (-20%)	p=0.0113

Fig.2e	*n*	mean(% change)	median(% change)	Log-rank (vs control)
Control	74	61.18	70	-
tBH5mM	74	50.12 (-18.1%)	47 (-32.9%)	p=0.0009

Fig.S2a	*n*	mean(% change)	median(% change)	Log-rank (vs control)
Control	80	31.79	32	-
tBH2.5mM	80	38.05 (+19.7%)	38 (+18.8%)	p<0.0001
tBH5mM	80	39.91 (+25.5%)	38 (+18.8%)	p<0.0001
tBH10mM	80	36.36 (+14.4%)	38 (+18.8%)	p=0.0041

Fig.S2b	*n*	mean(% change)	median(% change)	Log-rank (vs control)
Control	68	36.44	39	-
tBH1mM	71	42.38 (+16.3%)	42 (+7.7%)	$p<0.0001$
tBH5mM	69	44.43 (+21.9%)	42 (+7.7%)	$p<0.0001$

Fig.S2c	*n*	mean(% change)	median(% change)	Log-rank (vs control)
Control	70	81.43	87	-
tBH1mM	70	96.04 (+17.9%)	97 (+11.5%)	$p<0.0001$
tBH5mM	70	94.41 (+15.9%)	97 (+11.5%)	$p<0.0001$

Fig.S2d	*n*	mean(% change)	median(% change)	Log-rank (vs control)
Control	74	61.43	54	-
tBH1mM	72	56.46 (-8.1%)	54 (0%)	$p=0.0077$
tBH5mM	75	60.88 (-0.9%)	54 (0%)	$p=0.5896$

Fig.S2e	*n*	mean(% change)	median(% change)	Log-rank (vs control)
Control	71	45.27	47	-
tBH1mM	67	46.73 (+3.2%)	47 (0%)	$p=0.1473$
tBH5mM	77	51.48 (+13.7%)	47 (0%)	$p=0.0005$

Fig.S2f	*n*	mean(% change)	median(% change)	Log-rank (vs control)
Control	68	159.53	168	-
tBH2.5mM	69	168 (+5.3%)	168 (0%)	$p=0.0685$
tBH7.5mM	69	166.26 (+4.2%)	168 (0%)	$p=0.0843$

Fig.4e	*n*	mean(% change)	median(% change)	Log-rank (vs control)
Control	175	51.5	53	-
tBH0.5mM	168	56.89 (+10.5%)	59 (+11.3%)	$p<0.0001$
G418 25mg/L	171	57.78 (+12.2%)	59 (+11.3%)	$p<0.0001$

Fig.5d	*n*	mean(% change)	median(% change)	Log-rank (vs control)
Control	209	51.16	52	-
tBH5mM at F0	198	56.37 (+10.2%)	57 (+9.6%)	$p<0.0001$

Fig.S5d	*n*	mean(% change)	median(% change)	Log-rank (vs control)
Control	188	55.23	57	-
tBH5mM	197	48.52 (-12.1%)	48 (-15.85%)	$p<0.0001$
G418 100mg/L	192	41.52 (-24.8%)	39 (-31.6%)	$p<0.0001$

Fig.6c	*n*	mean(% change)	median(% change)	Log-rank (vs control)
Control	78	45.47	44	-
Control-RTA	82	60.12 (+32.2%)	58 (+31.8%)	$p<0.0001$
Control-MVNTA	75	59.64 (+31.2%)	58 (+31.8%)	$p<0.0001$
tBH5mM	79	53.32 (+17.3%)	52 (+18.2%)	$p<0.0001$
tBH5mM-RTA	79	57.8 (+27.1%)	58 (+31.8%)	$p<0.0001$<br>$p=0.1075$ (vs tBH)
tBH5mM-MVNTA	84	57.81 (+27.1%)	58 (+31.8%)	$p<0.0001$<br>$p=0.0489$ (vs tBH)

Fig.6d	*n*	mean(% change)	median(% change)	Log-rank (vs control)
Control	151	58.55	61	-
Control-RTA	151	71.31 (+21.8%)	79 (+29.5%)	$p<0.0001$
Control-MVNTA	153	69.2 (+18.2%)	76 (+24.6%)	$p<0.0001$
tBH5mM	144	62.6 (+6.9%)	64 (+4.9%)	$p<0.0001$
tBH5mM-RTA	150	67.76 (+15.7%)	73 (+19.7%)	$p<0.0001$<br>$p<0.0001$ (vs tBH)
tBH5mM-MVNTA	152	63.73 (+8.8%)	67 (+9.8%)	$p<0.0001$<br>$p=0.3526$ (vs tBH)

Fig.6e	*n*	mean(% change)	median(% change)	Log-rank (vs control)
Control	139	52.22	56	-
Control-RTA	143	56.93 (+9.0%)	65 (+16.1%)	p<0.0001
tBH 5mM	144	59.37 (+13.7%)	65 (+16.1%)	p<0.0001
tBH 5mM-RTA	139	62.82 (+20.3%)	68 (+21.4%)	p<0.0001<br>p=0.0054 (vs tBH)

Fig.7e	*n*	mean(% change)	median(% change)	Log-rank (vs control)
Control	161	51.6	51	-
Control-FO1	149	52.67 (+2.1%)	54 (+5.9%)	p=0.0229
tBH5mM	155	55.39 (+7.3%)	54 (+5.9%)	p<0.0001
tBH5mM-FO1	158	51.9 (+0.6%)	51 (0%)	p=0.3497<br>p<0.0001(vs tBH)

Fig.7f	*n*	mean(% change)	median(% change)	Log-rank (vs control)
Control	178	56.9	57	-
Control-FO1	183	58.55 (+2.90%)	60 (+5.26%)	p=0.0171
Control-FO2	175	56.58 (-0.56%)	57 (0%)	p=0.5587
PQ 1mM	177	64.94 (+14.1%)	66 (+15.8%)	p<0.0001
PQ 1mM-FO1	175	54.92 (-3.48%)	57 (0%)	p=0.132<br>p<0.0001 (vs PQ)
PQ 1mM-FO2	152	63.73 (+8.8%)	67 (+9.8%)	p=0.993<br>p<0.0001 (vs PQ)
